# ANLN: A New Hub in Glutamine Metabolism of Lung Adenocarcinoma by scRNA-Seq and Machine Learning

**DOI:** 10.32604/or.2026.079515

**Published:** 2026-07-16

**Authors:** Yiming Ma, Zhihan Zhang, Hongli Pan, Hailin Jiang, Lili Guo, Fengjie Guo

**Affiliations:** 1 The South China University of Technology School of Medicine, Guangzhou, China; 2 Center for Pancreatic Cancer Research, The South China University of Technology School of Medicine, Guangzhou, China; 3 Tianjin Key Laboratory of Lung Cancer Metastasis and Tumor Microenvironment, Tianjin Lung Cancer Institute, Tianjin Medical University General Hospital, Tianjin, China; 4 Precision Medicine Center, The Affiliated People’s Hospital of Shanxi Medical University, Taiyuan, China

**Keywords:** Lung adenocarcinoma, metabolism of glutamine, Single-cell RNA-seq, mast cells, ANLN, Prognostic model, tumor microenvironment

## Abstract

Objectives: Lung adenocarcinoma (LUAD) has a poor prognosis, and effective metabolic biomarkers are still few. Glutamine metabolism is one of the central features of tumor metabolic reprogramming, but the cellular heterogeneity and clinical significance of glutamine metabolism in the LUAD tumor microenvironment (TME) remain unknown. The goal of this paper was to define glutamine metabolism on a single-cell basis and determine major regulators that have predictive value. Methods: A single-cell RNA sequencing dataset (GSE149655) was combined with The Cancer Genome Atlas Lung Adenocarcinoma (TCGA-LUAD) and Gene Expression Omnibus (GEO) datasets in order to evaluate the metabolic activity and intercellular communication. The prognostic model was constructed on weighted gene co-expression network analysis (WGCNA), machine learning-based approaches, and least absolute shrinkage and selection operator (LASSO)-Cox regression to examine the characteristics of the immune system and sensitivity to drugs. Carried out CRISPR/Cas9 knockout experiments to prove the function of ANLN. Results: Glutamine metabolism activity and increased cell-cell communication were observed in mast cells. A gene signature of 4 genes (ANLN, CIP2A, MEST, WDR76) divided the patients into high-risk and low-risk groups; the survival of these two groups, immunosuppressant features of TME, and susceptibility to dasatinib were different. ANLN was also determined to be an essential prognostic driver, and downregulating it inhibited glutamine metabolism and the invasion properties of LUAD cells. Conclusions: Mast cells are metabolic centers of LUAD, whereas ANLN is a mediator of the association between glutamine metabolism and the development of tumors, which offers possible treatment options to achieve precise prognostication and treatment goals.

## 1 Introduction

Lung adenocarcinoma (LUAD) makes up at least 40–50% of all non-small cell lung cancer (NSCLC), and it is one of the main cell types of the condition; it is the main cause of cancer deaths worldwide [1,2]. Anatomically, LUAD largely derives its origin in the epithelial cells of the respiratory bronchioles and type II alveolar epithelial cells, with a small percentage of cases arising in the glands of mucus of large airways. Although lung cancer has traditionally been linked to smoking, LUAD has a different epidemiologic profile: it is the most commonly diagnosed subtype amongst nonsmokers and significantly affects females and Asian ethnicities. Nevertheless, it is not exclusive to such groups because clinical findings prove that it exists in a wide range of individuals, irrespective of age, ethnicity, and continent, including both Asians and Europeans. This particular histological subtype generally appears as a peripheral, round or oval mass, which poses special difficulties in early detection and pathological differentiation.

Despite advances in targeted therapy and immunotherapy, the prognosis of LUAD remains unsatisfactory, with a notable proportion of patients experiencing recurrence and metastasis even after curative resection. As one example, Reck et al. examined data on a phase III clinical trial and found that first-line immunotherapy showed minimal long-term survival effect in advanced LUAD [3]; Kim et al. listed all outcomes where the rate of recurrence and metastasis is particularly high, even after undergoing curative resections [4]. The five-year survival rate, which is almost treated as the gold standard in the prognosis of cancer, continues to get worse with every day passing if such words were to come after our later-stage diagnosis. Although surgery removes the tumor, the likelihood of recurrence and metastases in patients with earlier stage diagnoses remains alarmingly high, frequently higher than in other solid tumors [5]. The span of survival significantly varies, with numerous patients having a survival prediction of 3–5 years and relatively few patients surviving up to 5–10 years after diagnosis. This extreme recurrence and mortality rate demonstrate the ongoing weaknesses of the existing clinical practice and highlight the immediate need to look into new prognostic predictors and treatment goals.

Development of tumors and metastasis in LUAD is highly associated with metabolic reprogramming. And then do the isotope tracing of the metabolomics profiling, transcriptomics of this tumor has demonstrated how important glutamine metabolism is. For its bioenergetics, its redox potential, and biosynthesis, it was demonstrated by these types of experiments [4,5]. Glutamine acts as an essential carbon and nitrogen substrate utilized in making nucleotides, lipids, and amino acids, but also controls immune responses in the tumor microenvironment (TME) [6,7,8,9,10]. In more recent studies that have been conducted, uncontrolled glutamine utilization has been linked with the escape that is brought about by the immune system due to an impaired role of T-cells and the polarization that is induced towards immunosuppressive macrophages, which results in therapy resistance [11,12].

Nevertheless, there is no systematic knowledge of the mechanisms of how glutamine metabolic reprogramming precisely controls LUAD progression in the multifactorial TME. Although the newest single-cell RNA sequencing (scRNA-seq) technology is a promising breakthrough to enhance the quality of cellular diversity and its metabolic heterogeneity [13,14,15,16], the precise role of immune cell subpopulations playing a part in the metabolic landscape is almost entirely unexplored. Moreover, despite the existence of many transcriptomic studies identifying signatures of genes involved in glutamine metabolism, few strong prognostic models combining these signatures with machine learning in order to inform clinical behavior exist. It is important to address the gap between cellular metabolic heterogeneity and clinical practice.

To overcome these hurdles, we utilized an integrative multi-omics approach to elucidate the metabolic and molecular landscape of LUAD. First, utilizing scRNA-seq data, we systematically mapped the metabolic microenvironment of the TME and surprisingly discovered that mast cells represent the subpopulation with the highest glutamine metabolic activity. Second, our intercellular communication analysis demonstrated how these metabolically active mast cells interact with other cell types through signaling pathways such as epidermal growth factor (EGF) and vascular endothelial growth factor (VEGF). Third, employing Weighted Gene Co-expression Network Analysis (WGCNA) and enhanced machine learning algorithms, including support vector machine-recursive feature elimination (SVM-RFE) and Random Forest, we filtered out core genes to identify precise drivers of disease advancement. Fourth, we developed a powerful 4-gene prognostic model (comprising ANLN, CIP2A, MEST, and WDR76) that serves as an effective predictor of patient survival and correlates significantly with immune infiltration and sensitivity to drugs such as dasatinib. Finally, we experimentally validated the biological function of the core gene ANLN via knockdown assays, demonstrating its essential role in driving glutamine metabolism and promoting the proliferation, migration, and invasion of LUAD cells.

## 2 Materials and Methods

### 2.1 Data Acquisition

Transcriptome profiling data and corresponding clinical information of 513 LUAD tissues and 58 adjacent normal tissues were acquired from The Cancer Genome Atlas (TCGA) https://www.cancer.gov/ccg/research/genome-sequencing/tcga via the Genomic Data Commons (GDC) portal using the R package ‘TCGAbiolinks’ (version ‘2.38’). Additionally, two independent LUAD cohorts, GSE31210 (226 tumors, 20 normal tissues) and GSE30219 (85 tumors, 14 normal tissues), were downloaded from the Gene Expression Omnibus (GEO) database https://www.ncbi.nlm.nih.gov/geo/summary/?type=taxfull using the ‘GEOquery’ R package (version ‘2.78’). Probe IDs were matched to gene symbols based on the respective GPL platform annotation files. After removing duplicated and unannotated probes, a total of 29 target glutamine metabolism-related genes were identified from the Gene Set Enrichment Analysis (GSEA) molecular signatures database (MSigDB) https://www.gsea-msigdb.org/gsea/msigdb for downstream integrated analysis.

### 2.2 Methods of Processing and Analyzing Single-Cell RNA Sequencing Data

Single-cell RNA sequencing (scRNA-seq) data from the GSE149655 cohort were processed utilizing the ‘Seurat’ R package (version ‘4’). Stringent quality control was applied: cells with >10% mitochondrial gene expression or with nFeature_RNA outside the range of 200–3000 were excluded. The ‘Harmony’ algorithm was utilized to correct for batch effects across different samples. Following highly variable gene selection and Principal Component Analysis (PCA), the optimal principal components were determined via the Elbow method. Unsupervised cell clustering was performed using the FindClusters function (yielding 16 clusters) and visualized via Uniform Manifold Approximation and Projection (UMAP) [17,18]. Seven major cell subsets were annotated based on canonical cell-type markers from the literature. Intracellular glutamine metabolism (GM) activity at the single-cell level was quantified using the AddModuleScore function. Finally, differentially expressed genes (DEGs) between high- and low-score groups were identified [18,19,20]. Cells were stratified into high- and low-score groups based on the median value of the glutamine metabolism score (stored in Add1). Specifically, cells with a score greater than the median across all cells were assigned to the “score_UP” group, while cells with a score less than or equal to the median were assigned to the “score_DOWN” group. This median-based cutoff was applied to ensure a balanced comparison between the two groups. Add1 represents the glutamine metabolism score calculated by the AddModuleScore function from Seurat, which averages the expression levels of the user-defined gene set for each cell. The glutamine metabolism gene set is presented in Table S1: Glutamine metabolism.

### 2.3 Analysis of Cell-Cell Interactions

To infer intercellular communication networks from scRNA-seq data, the R package ‘CellChat’ (version 1.6.1) was employed. The signaling interactions between different cell types were evaluated, and the ligand-receptor interaction networks were visualized using circle plots and heatmaps, as previously described [21].

CellChat-specific preprocessing:

The CellChat database was subset to “Secreted Signaling” interactions using subsetDB (CellChatDB, search = “Secreted Signaling”).

Expression data were subsetted using subsetData (cellchat1).

Overexpressed genes and interactions were identified using identifyOverExpressedGenes and identifyOverExpressedInteractions.

Data were projected onto the human PPI network using projectData (cellchat1, PPI. human).

Communication probabilities were computed with computeCommunProb (cellchat1, raw.use = TRUE), and interactions were filtered to retain those occurring in at least 10 cells (filterCommunication (cellchat1, min.cells = 10)).

Circle plots were generated using netVisual_circle with parameters: vertex. weight = groupSize, weight.scale = TRUE, label.edge = FALSE. Heatmaps were generated using netAnalysis_signalingRole_heatmap with pattern = “all”, “outgoing”, or “incoming”, and netVisual_heatmap with signaling = “SPP1”, color.heatmap = “Blues”. Default CellChat parameters were used otherwise.

### 2.4 WGCNA

The ‘WGCNA’ R package (version 1.72) was utilized to construct a co-expression network and identify gene modules associated with lung adenocarcinoma (LUAD). The gene expression data used for WGCNA analysis were obtained from TCGA-LUAD (https://www.cancerimagingarchive.net/collection/tcga-luad/).

The expression matrix was transposed to a sample-by-gene format as required by the WGCNA package. Genes with variance below the 75th percentile were optionally excluded to reduce computational burden. Samples and genes with excessive missing values were removed using the goodSamplesGenes function. Outlier samples were detected via hierarchical clustering based on Euclidean distance and were manually excluded if present. No further normalization was applied, as the input data were pre-normalized (log_2_ [count + 1] transformation).

A soft-thresholding power of β = 20 was selected to ensure a scale-free topology. This value was chosen as the lowest power that achieved a scale-free topology fit index (R^2^) above 0.85 while maintaining a relatively high mean connectivity, as lower power values failed to meet the required R^2^ cutoff for the current dataset. A signed adjacency matrix was generated and transformed into a Topological Overlap Matrix (TOM). Gene modules were dynamically identified using hierarchical clustering and dynamic tree-cutting algorithms, with a minimum module size of 50 genes and a merge cut height of 0.3 to combine highly similar modules [22,23]. The modules were further correlated with clinical characteristics.

For each module, a module eigengene (ME) was calculated as the first principal component of the expression matrix for all genes within that module. Pearson correlation coefficients between each ME and each clinical trait were computed using pairwise complete observations. Corresponding *p*-values were calculated based on the Student’s *t*-distribution and were not adjusted for multiple testing, as the analysis was exploratory in nature. Modules with |correlation| ≥ 0.6 and *p* < 0.05 were considered significantly associated with the respective clinical trait. The module showing the highest absolute correlation with the target trait was identified as the key module. Hub genes within the key module were defined as those with module membership |MM| > 0.8 and gene significance |GS| > 0.5, and were subsequently subjected to GO and KEGG enrichment analyses using the clusterProfiler package (version 4.4.0).

### 2.5 Differential Gene Expression Analysis

The raw expression matrix was preprocessed as follows: expression values were log2(*x* + 1) transformed to reduce data skewness. Subsequently, between-array scale normalization was performed using the “normalizeBetweenArrays” function from the “limma” package to eliminate systematic differences between samples. To remove noise from low-expression genes, genes with a mean expression ≤ 1 across all samples were filtered out.

The ‘limma’ R package was used to identify DEGs between LUAD tumor and normal samples in the TCGA cohort. A total of 571 samples were included in the analysis, comprising 512 LUAD tumor tissues and 59 adjacent normal lung tissues. A linear model was fitted for each gene’s expression value, and an empirical Bayesian method (eBayes) was applied to moderately shrink the standard deviations. The criteria for defining DEGs were: |log2 fold change (log2FC)| > 1 (equivalent to a raw fold change > 2 or < 0.5) and an adjusted *p*-value < 0.05. Multiple testing correction was performed using the Benjamini-Hochberg (BH) method to control the false discovery rate (FDR) [24].

### 2.6 The GO Enrichments Analysis

Gene Ontology (GO) enrichment analysis, encompassing biological processes, molecular functions, and cellular components, was conducted using the ‘clusterProfiler’ R package (version 4.4.0) with the Over-Representation Analysis (ORA) approach. Human gene annotations were obtained from the ‘org.Hs.eg.db’ database (version 3.15.0, corresponding to GO release date 01 March 2022). The input gene list comprised core genes further screened from the brown module identified by WGCNA. The background gene set was defined as all genes belonging to the brown module (i.e., the complete gene set of that module). The key parameters used in the clusterProfiler:enrichGO function were as follows: pAdjustMethod = “BH” (Benjamini–Hochberg correction for multiple testing), pvalueCutoff = 0.05, and qvalueCutoff = 0.05. Only GO terms satisfying both criteria were considered statistically significant [25].

### 2.7 Set Enrichment Analysis (GSEA)

GSEA was performed using ‘clusterProfiler’ based on the ‘c2.cp.v7.2’ reference gene set from MSigDB. The analysis parameters were set as follows: random seed = 2020, permutations = 10,000, and gene set size limits = 10–500. A FDR < 0.05 was considered the threshold for significant enrichment [25].

### 2.8 Selection of Features through Machine Learning

To identify the most robust prognostic hub genes from the candidates, two machine learning algorithms were implemented: Random Forest (RF) and Support Vector Machine-Recursive Feature Elimination (SVM-RFE) [26]. For the RF algorithm, the ‘randomForest’ package (version 4.7-1.1) was employed with 500 trees (ntree = 500). The optimal number of trees was determined by identifying the point with the minimum out-of-bag (OOB) error rate. Feature importance was ranked using the mean decrease in accuracy (MeanDecreaseAccuracy) metric. For the SVM-RFE algorithm, a custom implementation based on the mSVM-RFE approach (John Colby, http://github.com/johncolby/SVM-RFE) was used with a linear kernel and a cost parameter set to 10. The recursive feature elimination process incorporated 5-fold cross-validation to determine the optimal feature subset. Specifically, the data were randomly partitioned into 5 folds, and features were recursively eliminated while computing weights across all folds. The optimal number of features was determined at the point minimizing the 5-fold cross-validation error, as implemented in the FeatSweep.wrap function. The cross-validation procedure was performed once (single iteration), as the recursive elimination process inherently evaluates feature stability.

### 2.9 Selection of Prognostic Factors and Development of Risk Scores

To eliminate multicollinearity among high-dimensional features, the least Absolute Shrinkage and Selection Operator (LASSO) Cox regression analysis was applied to the hub gene set. Prior to LASSO Cox regression analysis, each gene’s expression values were standardized across all samples to have a mean of 0 and a standard deviation of 1 (z-score transformation). The LASSO Cox regression model was then fit using the ‘glmnet’ package, with 10-fold cross-validation to select the optimal tuning parameter (lambda). The final risk score for each patient was calculated as the linear combination of the standardized gene expression values weighted by the LASSO coefficients. The prognostic risk score was calculated based on the linear combination of the expression levels of the optimized genes multiplied by their respective LASSO regression coefficients, using the following formula:


(1) $$RiskScore=\sum_{i=1}^{n}(Coefficient_{i}\times Expression_{i})$$

Regression coefficient, quantity of expression of each gene are shown as and respectively. In order to validate the predictive quality of the risk score as a prognostic parameter, this study has adopted the Kaplan-Meier survival analysis and then validated with an external independent validation cohort (GSE31210, GSE30219) [23].

The area under the curve (AUC) was calculated at specific time points (e.g., 1-, 3-, and 5-year) to assess the discriminative ability of the risk score for patient survival. Additionally, the concordance index (C-index) was computed using the ‘survcomp’ package to quantify the overall predictive accuracy of the prognostic model. A higher C-index (ranging from 0.5 to 1.0) indicates better predictive performance. Kaplan–Meier survival analysis was performed to compare overall survival between the high-risk and low-risk groups. The log-rank test was used to assess the statistical significance of the survival differences between the two groups.

### 2.10 Building and Testing the Prognostic Model

Univariate Cox regression analysis was performed to evaluate the prognostic value of the risk score and traditional clinical variables (e.g., age, gender, T stage, N stage, and overall stage) in the TCGA-LUAD cohort. Based on clinical relevance and established prognostic significance in lung adenocarcinoma, all variables were included a priori for nomogram construction to predict 1-, 2-, and 3-year overall survival, regardless of their statistical significance in univariate analysis. A multivariable Cox proportional hazards model was fitted using the ‘cph()’ function from the ‘rms’ package (version 6.3-0). The predictive accuracy and discriminative capability of the model were validated using time-dependent Receiver Operating Characteristic (ROC) curves and calibration plots [27].

### 2.11 How to Analyze the TME Using ESTIMATE

To evaluate the TME of the TCGA-LUAD cohort, we utilized the ‘estimate’ R package. This algorithm infers the fraction of stromal and immune cells in tumor samples based on gene expression data. Specifically, ImmuneScore, StromalScore, and ESTIMATEScore were calculated for each sample. These scores were then systematically compared between the high-risk and low-risk groups to assess differences in TME composition [28]. To compare the ImmuneScore, StromalScore, and ESTIMATEScore between the high-risk and low-risk groups, the Wilcoxon rank-sum test (Mann-Whitney U test) was employed. This non-parametric test was chosen because the score distributions did not conform to normality based on the Shapiro-Wilk test (*p* < 0.05). An FDR-adjusted *p*-value < 0.05 was considered statistically significant.

### 2.12 Analysis of Immune Cells Infiltration with CIBERSORTx

CIBERSORT (R package) was employed to estimate the relative proportions of 22 immune cell subtypes (LM22 signature matrix). The analysis was performed with the following parameters: permutation number = 1000 for significance testing, quantile normalization (QN = TRUE) applied to the expression data, and default signature matrix settings (LM22). The support vector regression (SVR) algorithm was used for deconvolution.

The input expression data were preprocessed as follows: (1) Data distribution was assessed by quantile analysis to determine the necessity of log2 transformation. Log2 transformation was applied if the 99th percentile exceeded 100, or if the data range exceeded 50 with a positive 25th percentile, or if the 25th percentile was between 0 and 1 while the 75th percentile exceeded 1. (2) Values ≤ 0 were set to NaN prior to log2 transformation to avoid undefined results. (3) Between-array quantile normalization was performed using the normalizeBetweenArrays function from the limma package to reduce technical variation between samples. (4) No additional batch correction was applied as all samples originated from the same TCGA-LUAD cohort.

### 2.13 Drug Sensitivity Analysis

Pharmacogenomic data were obtained from the Genomics of Drug Sensitivity in Cancer (GDSC2) database (https://www.cancerrxgene.org). Drug sensitivity prediction was performed using the oncoPredict R package, with the GDSC2 training dataset as the reference. The half-maximal inhibitory concentration (IC50) values of common anticancer drugs were predicted and log-transformed for analysis. We then systematically compared the IC50 values between the high-risk and low-risk groups to evaluate the correlation between the prognostic signature and drug sensitivity [29]. Patients were divided into high-risk and low-risk groups according to the risk scores derived from the preceding statistical analysis of clinical characteristics. The Wilcoxon rank-sum test was used to compare IC50 values between the two groups, and only drugs with a *p*-value less than 0.05 were considered significantly different. Multiple testing correction (e.g., FDR) was not applied in this analysis, as the *p*-value cutoff was set to an extremely stringent threshold (*p* < 1 × 10^−^^24^) for drug screening. Additionally, the association between the prognostic signature and drug sensitivity was further evaluated using Pearson correlation analysis between the risk score and the predicted IC50 values. The number and names of anticancer drugs included in the analysis are provided in the ‘DrugPredictions’ file in the supplementary materials Table S2 DrugPredictions.

### 2.14 Immunohistochemical Staining

The tissue microarray (TMA) used in this study was obtained from Shanghai Outdo Biotech Co., Ltd. (Shanghai Biobank, Shanghai, China) with catalog number HLugA060PG03 and lot number XT24-015. The TMA contained 60 cores from 30 patients with lung adenocarcinoma, including 30 tumor cores and 30 paired adjacent non-cancerous lung tissue cores. Each core had a diameter of 2.0 mm. The array layout consisted of 7 rows (A–G) and 10 columns (1–10), with one localization point (G01). Clinical and pathological characteristics of the cohort are summarized in Table S3. Briefly, the cohort comprised 15 males and 15 females (sex ratio 1:1), with an age range of 38–83 years (median: 64 years). Tumors were histopathologically classified according to the WHO classification, including grades I (n = 4), II (n = 10), and III (n = 16). Detailed clinicopathological parameters, including tumor size, lymph node metastasis, pleural invasion, and vascular invasion, are provided in Supplementary Table S4.

Tissue microarray sections were baked, deparaffinized, and rehydrated through a graded alcohol series according to the manufacturer’s instructions. Hematoxylin and eosin (H&E) staining was performed using a commercial kit (CAT# C0105M, Beyotime, Shanghai, China). For immunohistochemistry (IHC), antigen retrieval was performed in sodium citrate buffer. For antigen retrieval, the TMA sections were immersed in 10 mM sodium citrate buffer (pH 6.0) and heated in a pressure cooker at 121°C for 3 min. The sections were then allowed to cool naturally at room temperature for 20 min before proceeding with blocking steps. Endogenous peroxidase activity was blocked with 3% hydrogen peroxide for 10 min at room temperature (RT). Nonspecific binding was blocked using 5% goat serum (Solarbio, SL038, Beijing, China) at room temperature for 60 min. Sections were incubated overnight at 4°C with a primary antibody against ANLN (1:200; Boster Biological Technology, Pleasanton, CA, USA, CAT# A03997-1), followed by detection using an enzyme-labeled 3,3′-Diaminobenzidine (DAB) substrate. According to the manufacturer, this antibody recognizes human ANLN and has been validated for immunohistochemistry (IHC), Western blot (WB), and flow cytometry (FCM) applications. It was supplied as a lyophilized powder with a concentration of 0.5–1 mg/mL upon reconstitution. Following primary antibody incubation, the sections were incubated with a HRP-conjugated goat anti-rabbit IgG (H + L) secondary antibody (Proteintech Group, Rosemont, IL, USA; Catalog No.: SA00001-2). The secondary antibody was diluted at 1:10,000 in phosphate-buffered saline containing 1% bovine serum albumin (PBS/BSA). (PBS, Gibco, C10010500BT, Waltham, Massachusetts, USA) (BSA, Solarbio, 9048-46-8, Beijing, China) Incubation was performed at room temperature for 60 min. The DAB working solution should be prepared fresh and used immediately. Incubation for color development should be performed at room temperature under light-protected conditions, with the development time controlled within 1 min. Stained slides were digitized using a digital pathology scanner (Leica Aperio LV1, Leica Biosystems Imaging, Inc., Vista, CA, USA; manufactured in Germany). Immunostaining intensity was quantified using ImageJ Java 13.0.6 (National Institutes of Health, Bethesda, MD, USA; Laboratory for Optical and Computational Instrumentation, University of Wisconsin-Madison, Madison, WI, USA) with the IHC toolbox plugin. Statistical analyses were performed in GraphPad Prism 10 (GraphPad Prism Software Inc, LLC, San Diego, CA, USA) using Student’s *t*-test [30]. IHC image quantification method is a semi-automated, pixel-intensity-based positive area analysis using the “H-DAB” color deconvolution model from the IHC Toolbox plugin. The macro first separates the brown DAB-positive signal from the hematoxylin counterstain, then applies an automatic “Default” threshold to generate a binary mask representing the positive regions. Before measurement, it performs an uncalibrated optical density (OD) calibration, converting raw grayscale values using the formula OD = log_10_(255/pixel value). Finally, it measures both the Area (the number of pixels covered by positive signals) and the Integrated Density (IntDen) (the sum of OD values within the positive regions). The output data is saved as a CSV file, while processed binary images are saved as TIFF files.

### 2.15 Cell Culture and Transfection

Human lung adenocarcinoma cell lines H661 and H1299 were obtained from the American Type Culture Collection (ATCC, Manassas, VA, USA). H661 (ATCC^®^ HTB-177™) and H1299 (ATCC^®^ CRL-5803™) were cultured in RPMI-1640 medium (Gibco, C11875500BT, Waltham, Massachusetts, USA) supplemented with 10% fetal bovine serum (FBS) (Nobimpex GmbH, A115-500, Herbolzheim, Baden-Württemberg, Germany) and 1% penicillin-streptomycin (Gibco, 15140122, Waltham, Massachusetts, USA). Cells were maintained in a humidified incubator at 37°C with 5% CO_2_. Cell line authentication was performed by short tandem repeat (STR) profiling, and all cells were confirmed to be negative for mycoplasma contamination.

H661 is a non-small cell lung cancer (NSCLC) cell line that has also been used as a complementary model for lung adenocarcinoma research. Specifically, Tao et al. used H661 cells to investigate lymphangiogenesis in LUAD [31]; Nian et al. characterized NCI-H661 as a “human lung adenocarcinoma cell line” in studies on the PI3K/AKT/mTOR signaling pathway [32]. Therefore, we selected H661 as a complementary cell model for this study, alongside the canonical LUAD cell line H1299, to validate and extend our findings regarding glutamine metabolism in LUAD.

For transfection, cells were seeded at a density of 1 × 10^5^ cells per well in 6-well plates and grown to 70%–80% confluence before transfection. Transient transfection was performed using the Lipofectamine 3000 reagent (Thermo Fisher Scientific, Inc., CAT# L3000015, Waltham, MA, USA) according to the manufacturer’s protocol. Briefly, 2 μg of plasmid DNA was diluted in 125 μL of Opti-MEM and 5 μL of Lipofectamine 3000 was diluted separately in another 125 μL of Opti-MEM. The two solutions were diluted in Opti-MEM, mixed, and gently incubated for 10 min at room temperature to form DNA-lipid complexes. The mixture was then added to the cell culture. After 6–8 h of incubation at 37°C, the transfection medium was replaced with complete growth medium for subsequent experiments.

### 2.16 Glutamate Uptake Assay

Intracellular glutamate concentrations were determined using a glutamate assay kit (Solarbio, BC1580, Beijing, China) based on ultraviolet spectrophotometry. Cells were seeded at a density of 1 × 10^5^ cells per well in 6-well plates and cultured to 70%–80% confluence before transfection. At 24 h post-transfection, cells were serum-starved in serum-free medium for 12 h. At 36 h post-transfection, cells were incubated for an additional 12 h in medium supplemented with or without 2 mM L-glutamine. Cells were harvested and lysed ultrasonically in Reagent 1, followed by centrifugation at 9500× *g* for 10 min at 4°C. The supernatant was collected for detection. Glutamate concentrations were calculated from a standard curve according to the manufacturer’s instructions, with absorbance changes monitored at 340 nm using an ultraviolet spectrophotometer. (a NanoDrop One/Ultra spectrophotometer, Thermo Fisher Scientific, Waltham, MA, USA) Glutamate levels were normalized to cell number (per 10^6^ cells). All data were analyzed using GraphPad Prism 10 software (GraphPad Prism software Inc.). One-way ANOVA was used for intergroup comparisons, and *p* < 0.05 was considered statistically significant [33].

### 2.17 Glutamine Uptake Assay

Intracellular glutamine levels in cell lysates were measured using a glutamine colorimetric assay kit (Elabscience, E-BC-K853-M, Wuhan, China). Cells were seeded at 1 × 10^5^ cells per well in 6-well plates and cultured to 70%–80% confluence prior to transfection. At 24 h post-transfection, cells were serum-starved for 12 h. At 36 h post-transfection, cells were cultured overnight in medium with or without 2 mM L-glutamine. Cells were harvested, lysed, and filtered through a 50 KD ultrafiltration tube. Absorbance was measured at 450 nm using a microplate reader, and glutamine concentrations were calculated based on a standard curve. Glutamine levels were normalized to cell number (per 10^6^ cells). All data were analyzed using GraphPad Prism 10 software. One-way ANOVA was performed for statistical analysis, and *p* < 0.05 was considered statistically significant [34].

### 2.18 CCK-8 Assay

(1) Cell Seeding: H661 and H1299 cells at the logarithmic growth phase were digested with trypsin, resuspended, and counted. Cells were seeded into 96-well plates at a density of 3 × 10^3^ cells per well with 100 μL of medium containing 10% FBS (Nobimpex GmbH, A115-500). The plates were pre-incubated for 12 h at 37°C in a 5% CO_2_ incubator before transfection.
(2) Plasmid Transfection: After cell adhesion, the culture medium was discarded. Transfection complexes were prepared using Lipofectamine™ 3000 reagent (Thermo Fisher Scientific, Inc., CAT# L3000015) according to the manufacturer’s instructions. Briefly, Solution A was prepared by diluting 1 μg of Cas9, sgRNA1, or sgRNA2 plasmid in 125 μL of Opti-MEM, followed by the addition of 5 μL of P3000 reagent and gentle mixing. Solution B was prepared by diluting 2.5 μL of Lipofectamine 3000 in 125 μL of Opti-MEM and incubated at room temperature for 5 min. Solutions A and B were gently mixed and incubated at room temperature for 15 min to form DNA–transfection reagent complexes.
(3) Transfection Procedure: The prepared transfection complexes (250 μL) were mixed with 3 mL of serum-free medium. After gentle pipetting, 100 μL of the mixture was added to each well of the 96-well plate. The plate was gently shaken back and forth for uniform distribution, and cells were returned to the incubator for further culture (except for Day 0).
(4) Post-Transfection Treatment: At 6–8 h post-transfection, the medium containing transfection complexes was removed and replaced with 100 μL of fresh antibiotic-free medium to reduce cytotoxicity. The positive control, sgRNA1, and sgRNA2 groups were cultured in 10% FBS medium supplemented with L-glutamine (2 mM), whereas the negative control group was maintained in 10% FBS medium without L-glutamine.
(5) CCK-8 Incubation and Absorbance Measurement: Following medium replacement at 6–8 h post-transfection, the culture medium was removed on Day 0, and 110 μL of a 10:1 mixture of complete medium and CCK-8 solution (Beyotime, C0038, Shanghai, China) was added to each well. After gentle mixing, cells were incubated for 1–2 h, and absorbance was measured using a Multimode Microplate Reader (BioTek Instruments, Inc., 800TS, Winooski, Vermont, USA). Cells were then cultured continuously for 1, 2, 3, 5, and 6 days. Before each measurement, the medium was discarded and replaced with the same CCK-8 working solution, followed by incubation for 1–2 h. After incubation, absorbance at 450 nm was measured using the same Multimode Microplate Reader (BioTek, 800TS), with measurement duration consistent with that on Day 0. Wells containing only medium and CCK-8 reagent (without cells) served as blank controls. Note: On Day 5, the medium for Day 6 detection was replaced with medium containing 2% FBS to prevent cell death.
(6) Data Analysis: Cell viability was calculated using the following formula: Viability (%) = [(ODtreatment − ODblank)/(ODcontrol − ODblank)] × 100. The maximum and minimum values were excluded, and the mean was calculated from the remaining four replicate wells. All experiments were performed independently in biological triplicate. Data are presented as mean ± standard deviation (SD). Glutamate levels were normalized to cell number (per 103 cells). Statistical analysis was performed using GraphPad Prism 10.0 software, and one-way ANOVA was used for comparisons among groups. *p* < 0.05 was considered statistically significant.

### 2.19 Wound Healing Experiments

Use a marker pen to draw a straight line through the center of three consecutive wells on the bottom of a 6-well plate. Then draw one line on each side of the central line. Take H661 and H1299 cells in the logarithmic growth phase, trypsinize, resuspend, and count. Seed the cells into 6-well plates at a density of 5 × 10^5^ cells per well (each well contains 2 mL of culture medium with 10% FBS). Place the plate in a 37°C, 5% CO_2_ incubator and pre-culture for 12–24 h until cell confluence reaches 70% for transfection. After cells adhere, aspirate the original medium. Use Lipofectamine 3000 reagent (Thermo Fisher Scientific, Inc., CAT# L3000015) and prepare the transfection complex according to the manufacturer’s instructions. Brief procedure:

Solution A: Dilute 1 μg of Cris9, sgRNA1, and sgRNA2 plasmids separately into 125 μL of Opti-MEM, then add 5 μL of P3000 reagent and mix gently.

Solution B: Dilute 2.5 μL of Lipofectamine 3000 into 125 μL of Opti-MEM and let stand at room temperature for 5 min.

Gently mix Solution A with Solution B and let stand at room temperature for 15 min to form DNA-transfection reagent complexes.

Add the prepared transfection complex (total volume 250 μL) dropwise and evenly into each well containing 1 mL of antibiotic-free, serum-free medium. Gently rock the plate back and forth to mix. Return the plate to the incubator for continued culture. 6–8 h after transfection, aspirate the medium containing the transfection complex and replace it with 2 mL of fresh complete medium to reduce toxicity. After 36 h, when cells have grown to 95%–100% confluence forming a dense monolayer, use a sterile 200 μL pipette tip. Holding the tip perpendicular to the bottom of the plate and aligned with the marked reference lines, make a straight scratch along the center line, then make additional scratches on the left and right sides of the center line. After scratching, aspirate the waste liquid, wash twice with PBS, and replace the medium. For Cris9(+), sgRNA1, and sgRNA2 groups, replace with medium containing 10% FBS supplemented with glutamine. For the Cris9(−) group, replace with medium containing 10% FBS without glutamine. Immediately capture images of the scratch area using an inverted microscope at 5× objective magnification (randomly select 6 fields of view per well). Return the plate to the incubator for continued culture. Capture scratch images at the same positions at 0 and 24 h. Use image analysis software (Fiji ImageJ with the Wound Healing size tool-ijm plugin) (version 2.9.0, https://fiji.sc, Madison, WI, USA) to analyze the acquired images. Calculate the relative scratch width or area at each time point. The migration rate or wound closure percentage is calculated using the following formula:

Wound Closure (%) = [(A_0_ − A_t_)/A_0_] × 100%

where A_0_ represents the scratch area (or average width) at 0 h, and A_t_ represents the scratch area (or average width) at *t* hours.

All experiments are independently repeated three times. Data are presented as mean ± standard deviation (mean ± SD). Statistical analysis is performed using GraphPad Prism 9.0 software. Comparisons between groups are analyzed using two-way ANOVA, with *p* < 0.05 considered statistically significant.

### 2.20 Transwell Assays

BD Matrigel (BD Matrigel™ Basement Membrane Matrix (standard, 10 mL, Cat# 354234) was purchased from BD Biosciences (San Jose, CA, USA) and was thawed overnight at 4°C and kept on ice prior to use. For the invasion assay, the Matrigel was diluted 1:8 with ice-cold serum-free culture medium. Then, 50–100 μL of the diluted Matrigel was added to the upper chamber of each Transwell insert (24-well plate, 8-μm pore size) and evenly spread to cover the entire membrane surface. The inserts were incubated at 37°C for 30 min to allow gelation. For the migration assay, no Matrigel was applied.

After the indicated transfection treatment, cells were harvested by trypsinization, centrifuged (e.g., 9500× *g* for 5 min), and resuspended in serum-free medium (RPMI-1640 medium, Gibco, C11875500BT, Waltham, Massachusetts, USA; DMEM High Glucose Medium, Gibco, C11995500BT, Waltham, Massachusetts, USA) to obtain a single-cell suspension. The cell concentration was adjusted to 5 × 10^4^ cells/mL. Subsequently, 200 μL of the cell suspension (containing 1 × 10^4^ cells) was gently added into the upper chamber of each Transwell insert. The lower chamber was filled with 500 μL of complete culture medium containing 10% fetal bovine serum (FBS) (Nobimpex GmbH, A115-500) as a chemoattractant.

The plates were incubated at 37°C in a 5% CO_2_ incubator for 12 h. After incubation, the inserts were removed, and the medium in the upper chambers was aspirated. Non-migrated or non-invaded cells on the upper surface of the membrane were gently wiped off with a moistened cotton swab. The cells that had migrated or invaded to the lower surface of the membrane were fixed with methanol for 15 min at room temperature, followed by staining with 0.1% crystal violet for 20 min. After washing with PBS to remove excess dye, the inserts were air-dried. Stained cells were observed and imaged under a light microscope (Nikon Eclipse Ci-L microscope, Nikon Corporation, Tokyo, Japan) in at least five randomly selected fields per insert. The number of cells that had penetrated the membrane was counted using ImageJ software, and statistical analysis was performed.

### 2.21 Western Blotting Detection

Take H661 and H1299 cells in the logarithmic growth phase, digest with trypsin, resuspend, and count. Seed the cells at a density of 5 × 10^5^ cells per well in 60 mm culture dishes (each well contains 4 mL of medium with 10% FBS). Place the culture plate in a 37°C, 5% CO_2_ incubator and pre-culture for 12–24 h until the cell confluence reaches 70%–80% before transfection.

After the cells adhere, discard the original medium. Use Lipofectamine 3000 reagent to prepare the transfection complex according to the manufacturer’s instructions. Brief steps are as follows: Solution A: Dilute 2 μg of Cris9, sgRNA1, and sgRNA2 plasmids separately in 250 μL of Opti-MEM, then add 10 μL of P3000 and mix gently. Solution B: Dilute 5 μL of Lipofectamine 3000 in 250 μL of Opti-MEM and let it stand at room temperature for 5 min. Gently mix Solution A and Solution B, and let stand at room temperature for 15 min to form the DNA-transfection reagent complex.

Add the prepared transfection complex (total volume 500 μL) dropwise and evenly into the cell culture wells, which have been replaced with 2 mL of antibiotic-free, serum-free medium. Gently rock the culture plate back and forth to mix. Return the cells to the incubator and continue culturing.

6–8 h after transfection, discard the medium containing the transfection complex and replace it with 4 mL of fresh medium to reduce toxicity. For Cris9 (+), sgRNA1, and sgRNA2, replace with medium containing glutamine and 10% FBS. For Cris9 (−), replace with medium containing 10% FBS but without glutamine.

After 72 h, collect H661 and H1299 cells, and wash three times with pre-chilled PBS. Add 300 μL of RIPA Lysis Buffer containing protease inhibitors and phosphatase inhibitors to each well, and lyse on ice for 30 min. Use a cell scraper to collect the lysate, centrifuge at 4°C and 12,000× *g* for 15 min. Collect the supernatant, measure the protein concentration using a BCA Protein Assay Kit (Enhanced) (Beyotime Biotechnology, P0010, China), and adjust all sample concentrations to be consistent based on the results. Add 1/4 volume of 5× SDS-PAGE protein loading buffer relative to the RIPA volume, boil at 100°C for 15 min to denature the proteins, and then store at −20°C.

Take 10 μL of each protein sample (equal amounts) and perform 10% SDS-PAGE vertical electrophoresis using an electrophoresis apparatus. Run the stacking gel at a constant voltage of 80 V for 30 min. After the bromophenol blue indicator enters the separating gel, switch to 120 V and continue electrophoresis for 60 min until the bromophenol blue reaches the bottom of the gel.

After electrophoresis, use a wet transfer method to transfer the proteins from the gel to a PVDF membrane. The transfer conditions are as follows: constant current of 300 mA for 95 min. Immerse the membrane in TBST buffer containing 5% non-fat milk and block on a shaker at room temperature for 2 h. Incubate the membrane with the primary antibody ANLN diluted in antibody dilution buffer at the specified ratio (1:1000) on a shaker at 4°C overnight. The primary antibody information is as follows: ANLN (UpingBio, Catalog No.: YP-mAb-18040, 1:1000). Wash the membrane three times with TBST buffer, 5 min each time. Then incubate with the corresponding HRP-labeled secondary antibody (mouse species-specific) (Proteintech, Catalog No.: SA00001-1, 1:10,000) at room temperature for 1 h. After incubation, thoroughly wash the membrane with TBST. Cover the front side of the membrane evenly with the luminescent substrate, react for 2 min, and then capture the image using an imager. Wash the membrane three times with TBST buffer, 5 min each time. Incubate the membrane with the primary antibody α-Tubulin diluted in antibody dilution buffer at the specified ratio (1:10,000) on a shaker at room temperature for 2 h. The primary antibody information is as follows: α-Tubulin (Proteintech, Catalog No.: 66031-1-Ig, 1:10,000). Wash the membrane three times with TBST buffer, 5 min each time. Then incubate with the corresponding HRP-labeled secondary antibody (species-specific) (Proteintech, Catalog No.: SA00001-1, 1:10,000) at room temperature for 1 h. After incubation, thoroughly wash the membrane with TBST. Cover the front side of the membrane evenly with the luminescent substrate, react for 15 s, and then capture the image using an imager (Analytik GmbH+Co. KG, Konrad-Zuse-Straße 1, 07745 Jena, Germany).

### 2.22 Statistical Analysis

All statistical analyses were performed using R software (version 4.4.0) and GraphPad Prism 9.0 or 10.0 (GraphPad Prism Software Inc.). Continuous variables with normal distribution were analyzed using the independent-samples *t*-test, while non-normally distributed data were analyzed using the Mann-Whitney U test. Categorical variables were compared using the *χ*^2^ test or Fisher’s exact test. Univariate Cox proportional hazards regression was used to identify prognostic factors. All statistical tests were two-sided, and *p* < 0.05 was considered statistically significant.

## 3 Results

### 3.1 The Single-Cell Transcriptomic Profiling and Functional Annotation in LUAD

**Figure 1:**
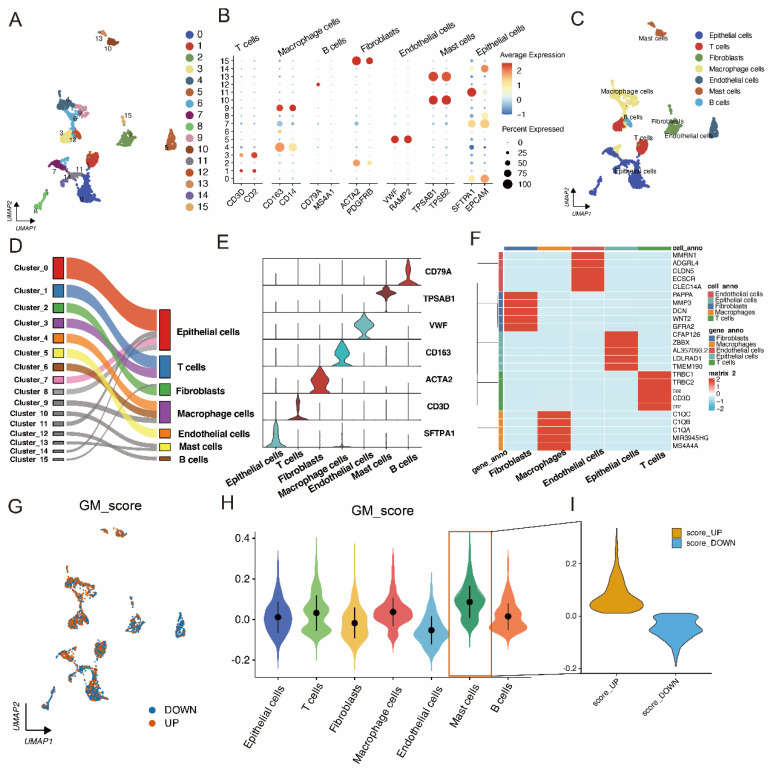
The single-cell landscape of lung adenocarcinoma (LUAD) indicates mast cells with peak values in glutamine metabolism (GM). (**A**) UMAP embedding of all high-quality single-cells of LUAD, coloured by unsupervised clusters. (**B**) Dot plot of canonical marker genes to annotate the cell-type; dot size reflects the proportion of cells expressing the given gene, and colour indicates average expression. (**C**) UMAP by the annotated cell types. (**D**) Sankey of unsupervised clusters mapped to ultimate cell-type annotations. (**E**) Expression of typical marker genes across annotated cell types using violin plots. (**F**) The top five differentially expressed genes per annotated cell type are displayed as a heatmap. (**G**) UMAP of per-cell GM activity scores (high-orange vs. low-blue), where the scores have been dichotomised based on a curated set of GM genes. (**H**) Distribution of GM activity scores by cell type; mast cells have the highest median GM activity. (**I**) Stratification of mast cells into two groups based on their median GM score: GM-high and GM-low, to perform further investigations.

To investigate the functional localization of glutamine metabolism during the development stage of lung adenocarcinoma and its relationship with tumor micro-environment, in this study, we systematically analyzed the single-cell transcriptomes of patients with lung adenocarcinoma. Using the UMAP dimensionality reduction clustering, we were able to find 16 subpopulations of cells that were highly differentially expressed (Fig. 1A). Depends on which of those marker gene expression profile (Fig. 1B), the aforementioned cell subpopulations were effectively partitioned into seven major cell phenotypes (Fig. 1C). Also, the association between first clustering clusters and final cell type labels was portrayed through a visual plot of a Sankey graph (Fig. 1D). The violin plot-based chart depicts that marker genes (Fig. 1E) are present in every kind of cell subtypes and a heatmap indicated the five most highly expressed genes per subtype as given in Fig. 1F.

To measure the level of GM activity, we applied the AddModuleScore function, which showed that there is considerable heterogeneity among various cells types (Fig. 1G). Mast cells had the highest GM scores (Fig. 1H). Subsequently, the study concentrates on MASTECELLS, which can be categorized into two types on the basis of their median scores (see Fig. 1I).

### 3.2 Communication between Cells and Pseudotemporality

**Figure 2:**
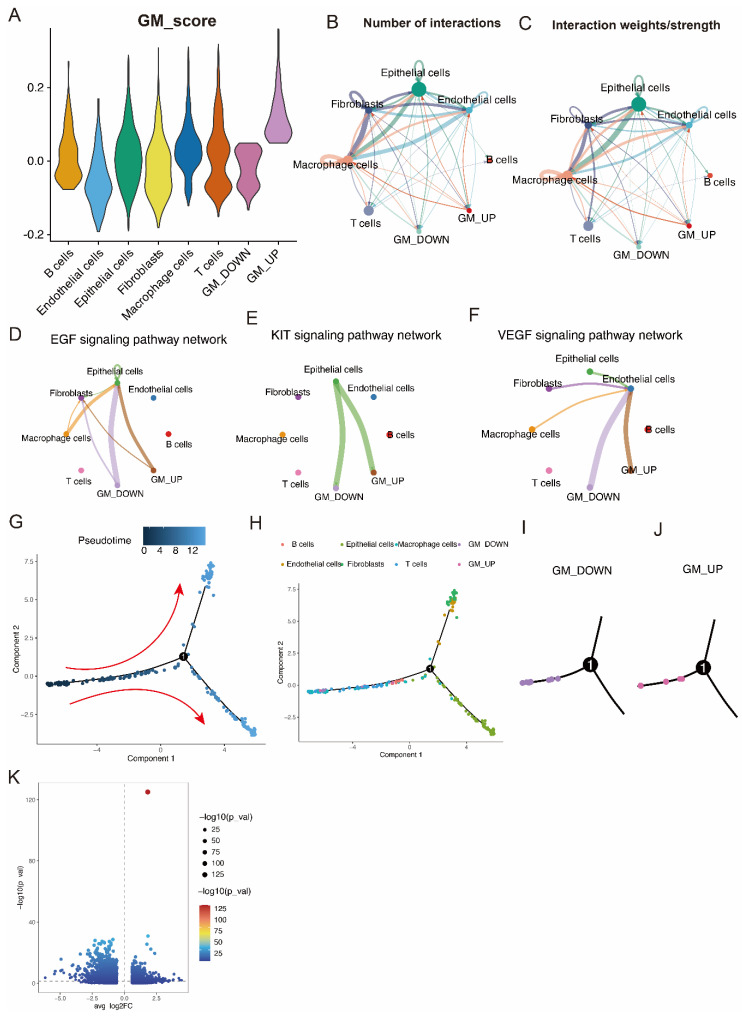
Mast cells with glutamine-metabolism (GM) scores demonstrate high levels of intercellular communication and the earliest pseudotime placement. (**A**) Stratifying mast cells by median GM score as High-GM or Low-GM subsets. Ligand-receptor interaction strength inferred by cellChat: (**B**) number of significantly interacting ligands and receptors, (**C**) the total interaction strength. EGF (**D**), KIT (**E**), and VEGF (**F**) pathway ligand-receptor signaling network; the edge thickness indicates the interaction strength, and the node size indicates the activity in the sender/receiver. (**G**) The pseudotime path of mast cells (arrows point at the predicted development between early and late stages). (**H**) The distribution of pseudotime on cells. The pseudotime distributions of High-GM (**I**) and Low-GM (**J**) subsets; lower pseudotime values represent more initial cellular states. (**K**) Differentially expressed genes (DEGs) between High-GM and Low-GM mast cells (volcano plot). The vertical dashed line indicates log_2_FC = 0, and the horizontal dashed line indicates −log_10_(*p*-value) = −log_10_(0.05).

Since GM-high mast cells are prominent, we also divided them according to the level of GM activity (Fig. 2A). As indicated in Fig. 2B,C, CellChat analysis showed that high-GM mast cells had a greater number and intensity of intercellular connections than low-GM cells. Cancer-relevant signaling pathways were enriched as specific pathways were examined (Fig. 2D–F), which included enrichment of EGF, KIT, and VEGF.

The pseudotime trajectory analysis defined the developmental path of cells (Fig. 2G,H). Most of the GM cells tended to be found in the earlier pseudotime levels (Fig. 2I,J), which could mean that GM plays a part in the initial tumor formation. The DE analysis between the GM groups revealed genes to be examined (Fig. 2K).

### 3.3 WGCNA Identifies a LUAD-Related Gene Module

**Figure 3:**
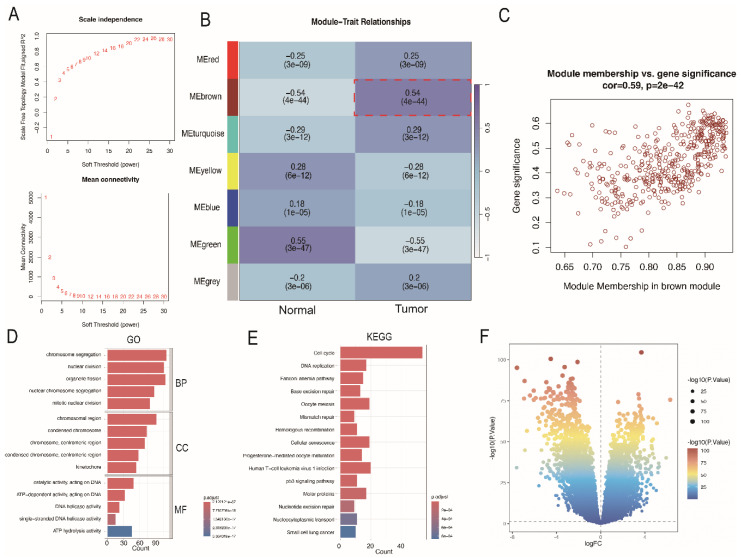
**Cell division is identified as a tumor-associated module by WGCNA.** (**A**) The soft thresholding power choice. Scale-free topology approximation was selected on soft-thresholding power equal to 20, retaining the network connectivity (see Methods). (**B**) Heatmap of module-trait correlations. In every cell are Pearson correlations between module eigengenes and tumor status, and the corresponding values are reported. The module that shows the highest positive correlation with tumor tissue is brown (as indicated by the red box). (**C**) Correlation between gene significance (GS) and tumor status and module membership (MM) in the brown module. Individual dots indicate individual genes, and the overall trend shows that there is a positive GS-MM correlation (correlation coefficient and *p* value shown). (**D**,**E**) Enrichment of brown-module gene functions. Analyses of gene ontology biological process (**D**) and Kyoto Encyclopedia of Genes and Genomes (KEGG) pathways (**E**) confirm the existence of significant enrichment of cell division/cell cycle-related terms. To control multiple testing, a false discovery rate (FDR) of the Benjamini-Hochberg was used, and only terms with less than 0.05 FDR were considered. (**F**) Expression difference between tumor and its neighboring normals (volcano plot) showing |log_2_ Fold Change (FC)| (*x*-axis) and −log_10_(*p*-value) (*y*-axis). Red and blue dots indicate highly expressed and lowly expressed genes, respectively (using predetermined cutoffs, see Methods). Where appropriate, representative brown-module genes are labeled. Red boxes and lines in this figure are explained as follows: B red box: Highlights the brown module with the highest positive tumor correlation. C red line: Linear regression fit between GS and MM.D/E red boxes/lines: Indicate significantly enriched cell-division-related terms (FDR < 0.05). F red lines: Represent log2FC and −log10(*p*-value) cutoffs for defining differentially expressed genes.

One of the main methods of the current research is to perform an analysis based on WGCNA using the data from the TCGA-LUAD database to identify the most significant genes. To obtain the best soft threshold that can be used to provide a scale-free network topology, this study chose the value of 20 (Fig. 3A). As per Fig. 3B, there are 7 co-expressed modules based on the analysis; one, the brown module, has a relatively high association with LUAD (correlation coefficient = 0.54). Also, the significance (GS) of the genes in this module is significantly correlated with their membership degree (MM) (Fig. 3C). Enrichment analysis showed links with chromosomal segregation, nuclear division, organelle fission, cell cycle, DNA replication, and other similar processes (Fig. 3D,E). By comparing the gene expression profiles of both tumor samples and normal samples, it was identified that certain specific genes were highly varying (Fig. 3F).

### 3.4 The Section below Will Be Based on the Selection of Key GM-Associated Genes Using Machine Learning

**Figure 4:**
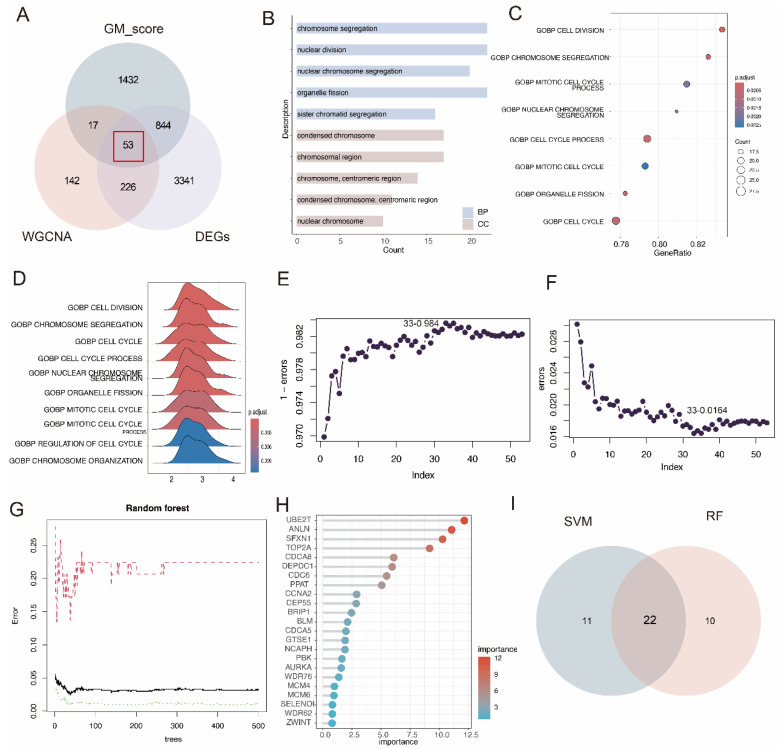
**The identification of 22 candidate genes that are related to glutamine metabolism (GM) using machine learning.** (**A**) Venn diagram of overlap between the genes included in the WGCNA brown module, tumour versus normal differentially expressed genes (DEGs), and GM-related DEGs (red box: n = 53 overlapping genes). (**B**) Gene ontology (GO) enrichment analysis of the 53 overlapping genes; the highest-ranking terms based on an adjusted *p*-value. The enrichment analysis of the core gene set as a dot-plot of enriched pathways (**C**) and a ridge plot of normalization enrichment scores (**D**). (**E**, **F**) Cross-validated error and number of features using SVM-RFE feature selection, including optimal subset. (**G**) Random forest (RF) out-of-bag error rate with the increasing number of trees. (**H**) Variable importance ranking (the top 15 genes) by the RF model. (**I**) Venn diagram of genes selected by the SVM-RFE and RF methods, with 22 shared core candidate genes (n = 22).

The integration of genes of the brown module, tumor differentially expressed genes (DEGs), and GM-related DEGs produced 53 candidate genes (Fig. 4A). Enrichment analysis of Gene Ontologies (GO) highlighted nuclear division and chromosome segregation procedures, among others (Fig. 4B). The results were also corroborated with Gene Set enrichment analysis (GSEA) (Fig. 4C,D). For achieving the best possible composition of candidate genomes, this paper used two machine learning approaches to filter out essential genes: Support Vector Machine (SVM; Fig. 4E,F) and Random Forest (RF; Fig. 4G) to filter important genes in 53 core candidate genes. The most significant 15 genes in the RF model can be presented using a dot-plot (Fig. 4H). Then, we have overlapped the gene sets chosen by SVM and RF to obtain 22 high-confidence genes (Fig. 4I).

### 3.5 Building and Testing the Predictive Risk Model

**Figure 5:**
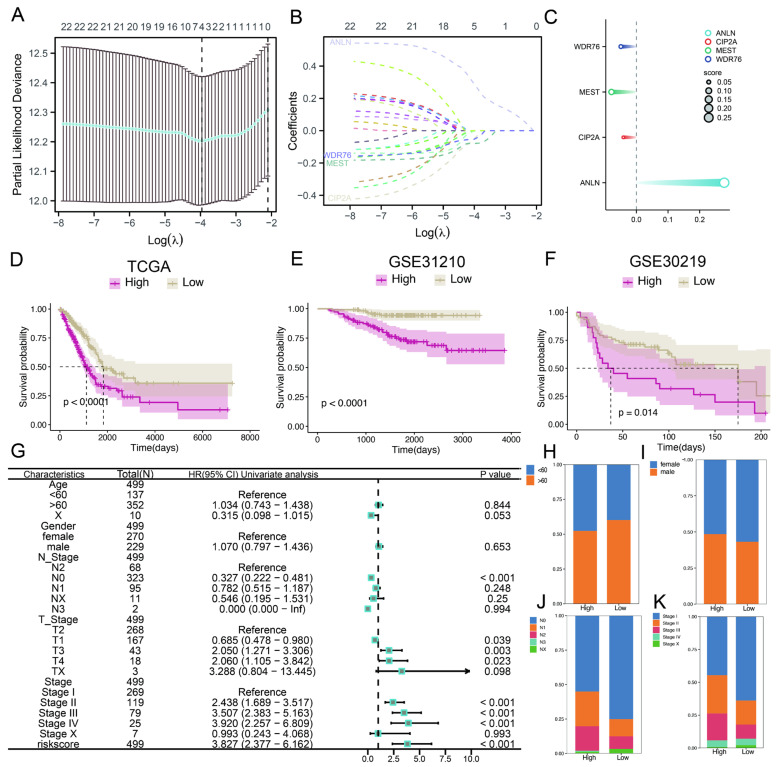
**The four-gene risk score is associated with total survival of LUAD in training and validation sets.** (**A**) Profiles of the LASSO Cox regression coefficients of the candidate genes in terms of the penalty parameter λ. (**B**) A cross-validation curve that was employed to determine the best choice of λ. (**C**) Final nonzero coefficients in the four-gene signature (ANLN, CIP2A, MEST, WDR76) model. Kaplan-Meier survival analysis on the comparison between high- and low-risk groups (median risk score cut-off) in TCGA (**D**), GSE31210 (**E**), and GSE30219 (**F**) with log-rank *p*-values indicated. (**G**) Univariate Cox regression (forest plot) reporting hazard ratios (HRs, 95 per cent CIs) based on the risk score plus clinical covariates. Distribution of clinical variables based on risk group: age (**H**), sex (**I**), N stage (**J**), and total stage (**K**).

The predictive genes were then used in performing the LASSO regression modeling in order to find the relevant gene markers and quantify the regression coefficients of the genes ANLN, CIP2A, MEST, and WDR76 (Fig. 5A–C). The expression levels of each gene multiplied and added together with their coefficients formed an individualized risk score per patient. So they were split up into two kinds: high risk and low risk. In Kaplan Meier survival analysis results, it can be seen that there is a difference in the overall survival rate between the high-risk and low-risk groups in TCGA (see Fig. 5D), GSE31210 (see Fig. 5E), and GSE30219 (see Fig. 5F) (all *p* < 0.05). Univariate Cox analysis was conducted to assess the effect of other clinical variables on overall survival in LUAD, and the results were displayed in a forest graph (Fig. 5G). The distributions of clinical features across risk groups were also shown (Fig. 5H–K), which showed that high N and total stages were correlated with worse survival.

**Figure 6:**
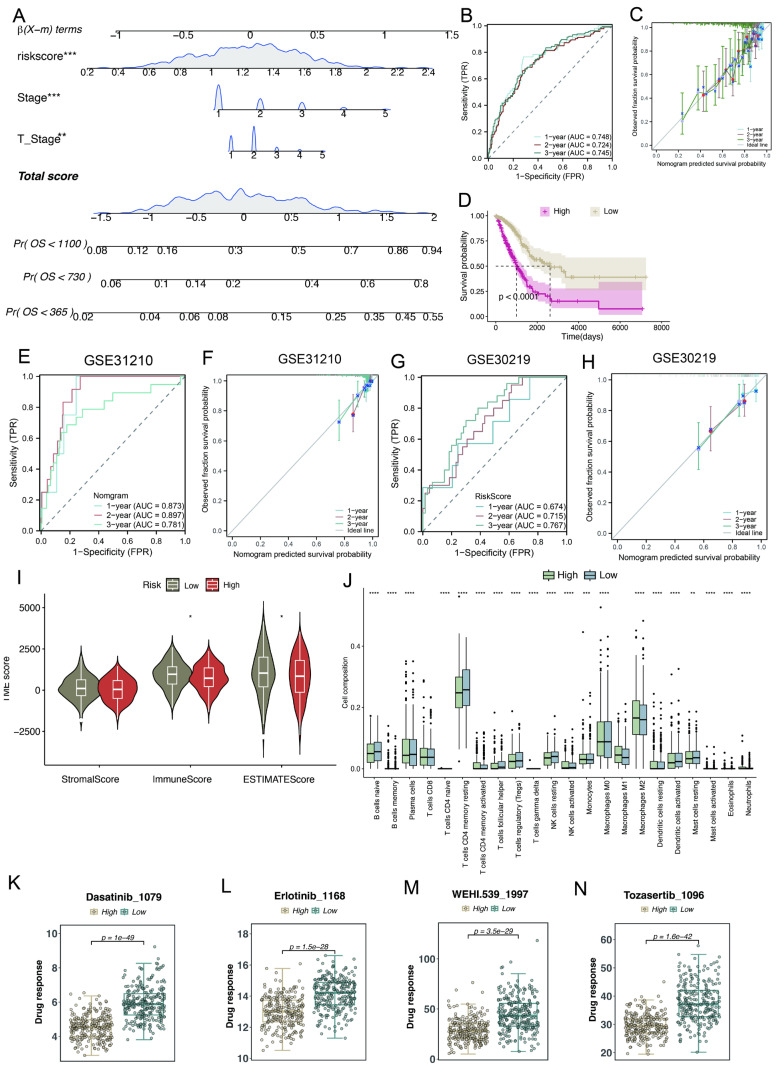
**Prognostic nomogram and correlations to tumor immunity and anticipated drug sensitivity.** (**A**) The nomogram combines a four-gene risk score with clinical covariate variables such as T stage and overall stage to estimate 1-, 2-, and 3-year overall survival (OS). Internal validation in TCGA: (**B**) time-dependent ROC curves at 1, 2, and 3 years, (**B**); (**C**) calibration plots comparing predicted and observed OS probabilities, (**C**); (**D**) Kaplan Meier (KM) curves of patients with OS divided by nomogram-derived risk groups. External validation in GSE31210: time-dependent ROC curves (**E**) and calibration plots (**F**). External validation in GSE30219: time-dependent ROC curves (**G**) and calibration plots (**H**). (**I**) Metrics of TME (ESTIMATE: stromal score, immune score, ESTIMATE score, and inferred tumor purity) based on the risk group. (**J**) Estimated relative immune cell infiltration by CIBERSORT in high vs. low-risk groups. (**K**–**N**) Drug predicted sensitivity by risk group is shown as half-maximal inhibitory concentration (IC50) of representative drugs. (**K**) Dasatinib, (**L**) Erlotinib, (**M**) WEHI-539, (**N**) Tozasertib. If not specified differently, *p*-values have been obtained on the basis of a two-sided test (e.g., log-rank test in case of KM analyses and Wilcoxon rank-sum in case of comparison between the groups). *p < 0.05; **p < 0.01; ***p < 0.001; ****p < 0.0001.

Using these findings, a nomogram (Fig. 6A) including risk scores and major clinical factors was plotted. To assess how reliable and precise this model is, we employ the TCGA data set to perform time-dependent ROCs, calibration plots, and Kaplan-Meier analysis (Fig. 6B–D). The performance of this nomogram has been consistent in two other outer validation groups, GSE31210 (Fig. 6E,F) and GSE30219 (Fig. 6G,H). This model is fairly precise in predicting 1, 2, and 3-year survivals, and the Area Under the Curve (AUC) is greater than 0.65. Also, the calibration curve indicates that the resulting predictive values in the model are very similar to the observed ones. These findings indicate that the model can be trusted and potentially applied in the future in the stratification of prognoses of LUAD.

We analyzed the correlation of the risk score with tumor immune microenvironment through the ESTIMATE algorithm which yielded high immune and ESTIMATE scores in high risk subjects (*p* < 0.05; Fig. 6I). We can tell this by when we are utilizing CIBERSORT and our analysis that we have a different number of infiltration for the other immune cells of each risk group (Fig. 6J) where there was an increased presence of M2 macrophages (high-risk group), indicating the possibility that immunosuppressive phenotypes may play a role in poor prognosis.

Also, we evaluated the relationship between risk score and drug effects from pharmacological response and gene data from the GDSC database. The differences in IC50 values among various risk groups with several chemotherapeutic agents, as well as targeted agents, were significant (*p* < 0.005). In particular, there was a heightened sensitivity of high-risk patients to dasatinib (Fig. 6K), erlotinib (Fig. 6L), WEHI-539 (Fig. 6M), and tozasertib (Fig. 6N).

### 3.6 ANLN as a Critical Factor of Prognosis in LUAD

**Figure 7:**
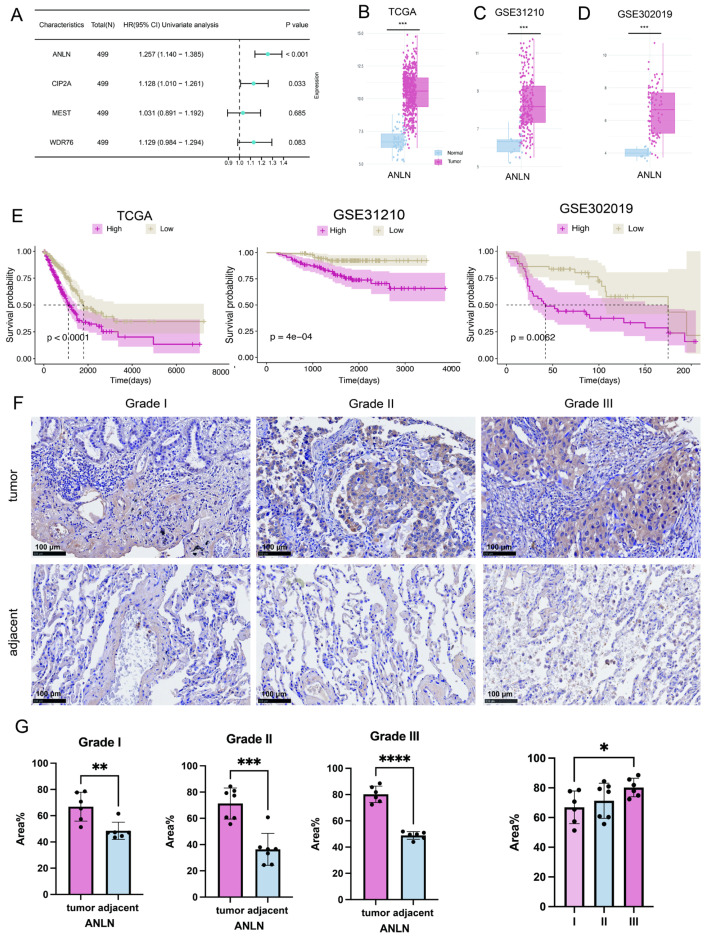
**Overexpression of ANLN and association with prognosis in lung adenocarcinoma (LUAD).** (**A**) The results of univariate Cox proportional hazards regression analysis of the four-gene panel (ANLN, CIP2A, MEST, WDR76) in the TCGA LUAD group is presented in a forest plot showing the hazard ratios (HRs) with 95% confidence intervals and *p*-values. ANLN mRNA expression in tumor vs. non-tumor lung tissues in TCGA (**B**), GSE31210 (**C**), and GSE30219 (**D**); these are depicted as relative expression, and the *p* values were calculated using a two-sided statistical test as described in the Methods. Overall survival analysis according to ANLN expression stratification (high vs. low, dichotomized based on the cohort-specific median) in TCGA, GSE31210, and GSE30219; ticks denote censored data, and *p*-values are obtained through the log-rank test (two-sided) (**E**). Human LUAD tissue microarrays were stained with ANLN with the help of immunohistochemistry (IHC). (**F**) Representative images showing comparison between tumor and adjacent non-tumor specimens with varying grades. (**G**) Quantitative analysis (Area %) ANLN expression in histological grade. *p < 0.05; **p < 0.01; ***p < 0.001; ****p < 0.0001.

Univariate Cox analysis was used to evaluate the prognostic value associated with the four core genes (ANLN, CIP2A, MEST, and WDR76) chosen in the LASSO regression based on TCGA data. ANLN was found to be the most statistically significant predictor of poor survival (Fig. 7A). The expression of the ANGN gene is much higher in cancerous tissues than in normal tissues, and this finding has been confirmed in the TCGA database, GSE31210, and GSE30219 databases (Fig. 7B–D). It is established that patients who have a high level of ANLN experience much lower total survival rates (Fig. 7E), therefore indicating that the ANLN gene could have some correlation in the genesis and progression of lung adenocarcinoma.

Moreover, immunohistochemical staining of tissue microarray revealed that the ANLN staining was higher in LUAD samples compared to normal adjacent tissue (Fig. 7F). Moreover, ANLN expression levels were positively correlated with higher tumor grades (grade II/III) (Fig. 7G), which supports its possible involvement as an oncogene.

### 3.7 The Validation of ANLN Functionality

**Figure 8:**
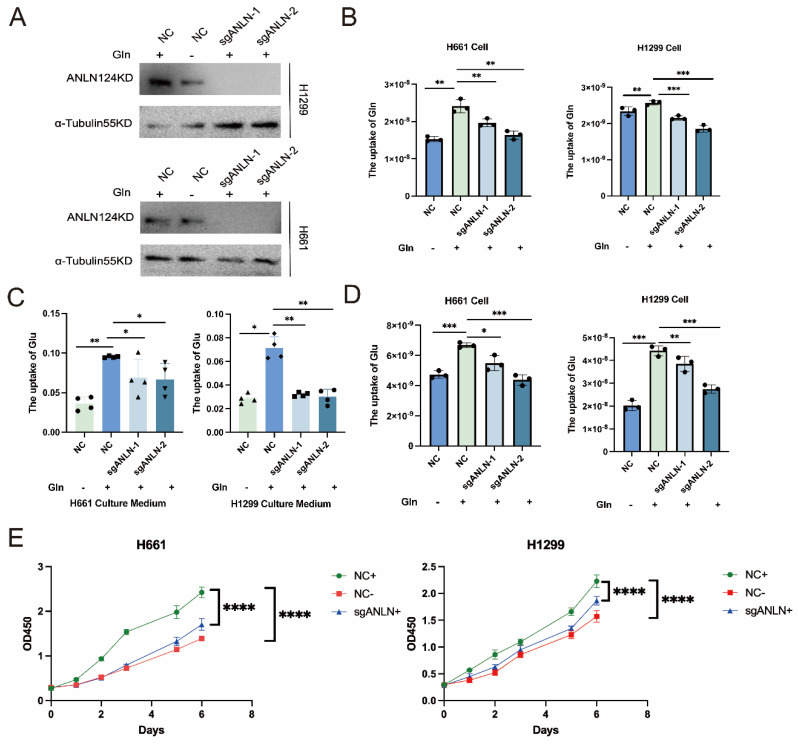
Depletion of ANLN results in the disruption of glutamine metabolism and inhibition of proliferation in LUAD cells. (**A**) ANLN protein expression was analyzed by immunoblotting after knockout with two independent sgRNAs (sgANLN-1, sgANLN-2) vs. a non-targeting control (NC) under glutamine-replete (+) or glutamine-free (−) conditions. Loading control was measured using alpha-tubulin. (**B**) Intracellular glutamine levels were measured 12 h post-treatment under the given conditions. (**C**) Measure Extracellular Glutamine Consumption after 12 h of treatment. (**D**) Glutamate levels were measured in the cell 12 h post-treatment. (**E**) Measurement of cell proliferation by the CCK-8 assay over time post ANLN knockout. (+) = glutamine replete and (−) = glutamine free media respectively. The data are the mean value of more than or equal to 3 independent experiments. ABBREVIATIONS: Gln, glutamine; Glu, glutamate. *p < 0.05; **p < 0.01; ***p < 0.001; ****p < 0.0001.

To test if ANLN contributes to tumor growth through control of glutamine metabolism, knockout (KO) cell lines of ANLN LUADs were created via CRISPR/Cas9 with two distinct sgRNAs (Fig. 8A). Western blot validated this knockout efficiency. Following that, we investigated the metabolic pathways of glutamine by quantifying intracellular and extracellular glutamine and glutamate levels of ANLN-knockout cells grown in glutamine-supplemented medium. Knocking out ANLN caused a substantial reduction of intracellular glutamine (Fig. 8B), which indicates an inability to uptake glutamine. Also, extracellular glutamate usage was reduced (Fig. 8C), and the levels of intracellular glutamate decreased significantly (Fig. 8D), implying a flaw in the process of glutaminolysis. The metabolic fingerprint of ANLN-knockout cells closely resembled that of wild-type cells cultivated in the absence of glutamine, which supported a crucial contribution of ANLN to glutamine metabolism.

**Figure 9:**
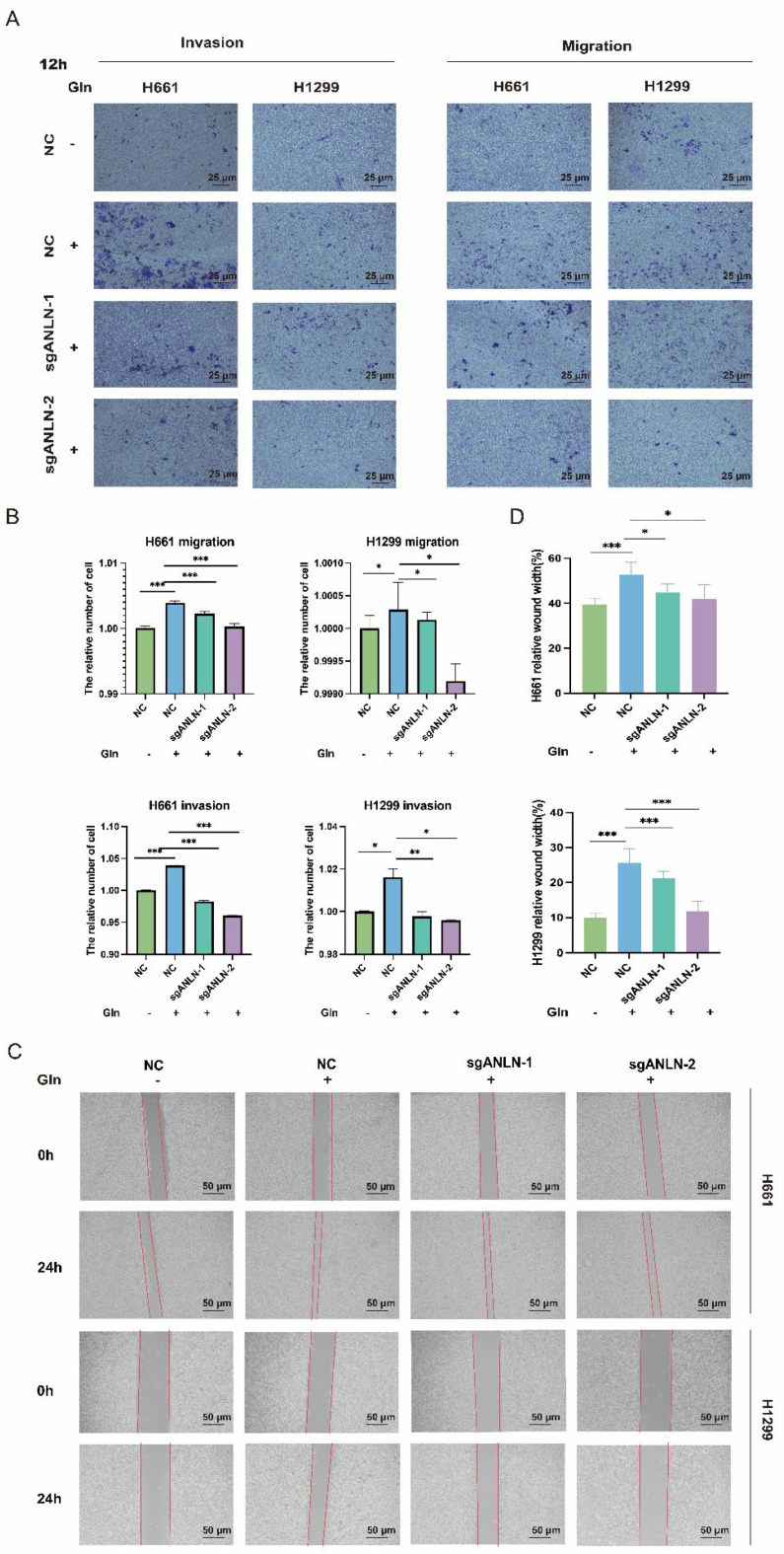
**Invasion and migration of LUAD cells are disrupted by an ANLN knockout.** (**A**) Images depicting Transwell invasion assays 24 h post ANLN knockout with two independent sgRNAs (sgANLN-1, sgANLN-2) or control. Scale bars = 25 μm. (**B**) Invaded/migrated cell counts quantified. (**C**) Pictures of wound-healing assays that were done right away (0 h) and 24 h after scratch under the conditions stated in this section. Scale bars = 50 μm. (**D**) The wound closure rate percentage was measured at 24 h in comparison to 0 h. (+) and (−) indicate glutamine-rich and glutamine-free medium, respectively. The numbers indicate mean + SD of at least three independent experiments, Gln: glutamine. *p < 0.05; **p < 0.01; ***p < 0.001.

We also tested the functionality consequences of ANLN absence. As measured by the CCK-8 assay, ANLN knockout considerably inhibited cell proliferation in 6 days (Fig. 8E). Wound healing, transwell invasion assays also showed the same in ANLN—knockout cells compared to control; there was a decrease in migration and invasion (Fig. 9A–D). It is shown that ANLN contributes to an increase in prolife proliferation, migration, and invasion of the malignancy phenotype of LUAD, with a partial role in glutamine metabolism.

## 4 Discussion

The current research reveals that glutamine metabolism plays a key part in the definition of the LUAD ecosystem, and mast cells become a surprise metabolic hub. Using our scRNA-seq, we have found that mast cells are the most metabolically active of all TME components, questioning their traditional role as merely immune regulators [35]. The fact that high-glutamine metabolism (GM) mast cells were enriched in early pseudotime suggests their possible participation in tumor initiation, which may occur by creating a metabolically permissive niche. This is in agreement with recent findings suggesting that mast cells play a role in early-stage tumourigenesis through the activation of stromal cells by cytokines [36,37]. Mast cells participate in the process of lung adenocarcinoma oncogenesis in the environment of the TME [38,39]. Nevertheless, the good prognostic significance of mast cells, rather than the unfavorable diagnosis, was also documented [40]. The detected up-regulation of EGF, KIT, and VEGF signaling pathways in GM-high mast cells further supports the role of the cells in both metabolic regulation and paracrine regulation [41], shedding new light on the mechanisms underlying how metabolic reprogramming and intercellular signals interact to promote LUAD progression.

On a molecular basis, our integrative omics strategy detected ANLN as one of the hub nodes connecting glutamine metabolism and aggressive tumors. ANLN is an actin-binding protein that participates in cytokinesis and has been previously found to be related to bad prognosis in many cancers, but no one has studied its metabolic roles [42,43,44,45]. In this paper, we will show that ANLN deletion is similar to glutamine starvation, reduces glutamate flux, and proliferative and invasive capacity. Such results put ANLN as a metabolic gatekeeper, which may regulate cytoskeletal reorganization in response to the presence of nutrients. ANLN expression levels are highly associated with tumor grade and also have a good predictive value among numerous cohorts, and this emphasizes its translational capabilities as a biomarker and therapeutic agent.

Our analysis showed that the four-gene risk model (ANLN, CIP2A, MEST, WDR76) had strong prognostic properties and performed better than traditional clinical factors in estimating survival. CIP2A, a well-known oncoprotein that stabilizes MYC, and MEST, a regulator of lipid metabolism, also support the cross-talk between glutamine metabolism and oncogenic signaling [46]. The presence of glutamine and its downstream products may also cause skewing of macrophage polarization toward M2 types and inhibit T-cell effector activities, which leads to immune evasion and drug-resistant cancer cells [12,47]. It is important to note that the immunosuppressive TME, including infiltration of M2 macrophages and increased expression of programmed death-ligand 1 (PD-L1), was observed among patients at high risk, as it can be expected based on the assumption that tumor metabolic wiring affects the actions of immune cells [48,49], indicating that the metabolic fluxes of glutamines might influence the mechanisms of immune escape. This is even more strengthened by the anticipated responsiveness of high-risk tumours to dasatinib and erlotinib, two tyrosine kinase inhibitors that can both cause immunomodulation.

Several limitations of this study should be acknowledged. Firstly, although our scRNA-seq results strongly support mast cell heterogeneity, spatial transcriptomics or multiplexed imaging are needed to better define their anatomical localization and intercellular communication. Secondly, the precise mechanistic link between ANLN and glutamine metabolism remains to be fully elucidated. Given its canonical role as an actin-binding protein, we speculate that ANLN might modulate glutamine metabolism by regulating the membrane trafficking, localization, or endocytosis of key glutamine transporters (such as ASCT2). Future experiments, such as surface biotinylation assays and co-immunoprecipitation (Co-IP), are warranted to evaluate the direct physical and regulatory interactions between ANLN and these transporters. Finally, the clinical utility of our 4-gene risk model requires large-scale prospective validation. In this context, exploring the model’s utility in liquid biopsies represents a highly promising future direction. Detecting the expression of this 4-gene signature in circulating tumor cells (CTCs) or tumor-derived exosomal RNA could significantly enhance its clinical applicability, offering a robust, non-invasive method for the real-time monitoring of disease progression and treatment response in LUAD patients.

Limitations regarding mast cell findings:

A notable limitation of this study is the lack of functional validation for mast cells. Although scRNA-seq identified GM-high mast cells as a distinct subpopulation with enhanced intercellular communication, we did not perform any loss- or gain-of-function experiments (e.g., mast cell depletion, GM gene knockout, or adoptive transfer) to establish causality. Consequently, the conclusion that mast cells act as “metabolic hubs” remains speculative at this stage. Our data should be interpreted as providing a prioritized set of hypotheses for future mechanistic investigation.

## 5 Conclusions

In conclusion, this study uncovers mast cells as a cell type with unexpectedly high glutamine metabolism gene expression in LUAD, and experimentally validates ANLN as a critical downstream effector linking glutamine metabolism to aggressive tumor behavior. As the prognostic model and therapeutic vulnerabilities described herein indicate, the future of precision oncology strategies that address the metabolic dependencies observed in LUAD is on its way. Further research ought to investigate whether the combination of ANLN inhibitor treatment and glutaminase inhibitors or immunotherapy will have synergistic effects on clinical outcomes in high-risk patients.

## Data Availability

The authors said that all data that supports the findings of this paper are as follows. Data openly available in a public repository. The data that support the findings of this study are openly available at: https://www.gsea-msigdb.org/gsea/msigdb/cards/GOBP_GLUTAMINE_METABOLIC_PROCESS.html, https://www.ncbi.nlm.nih.gov/geo/query/acc.cgi?acc=GSE3141, https://www.ncbi.nlm.nih.gov/geo/query/acc.cgi?acc=GSE30219, https://www.ncbi.nlm.nih.gov/geo/query/acc.cgi?acc=GSE31210, https://www.ncbi.nlm.nih.gov/geo/query/acc.cgi?acc=GSE72094, https://www.ncbi.nlm.nih.gov/geo/query/acc.cgi?acc=GSE149655.

## Supplementary Materials

The supplementary material is available online at https://www.techscience.com/doi/10.32604/or.2026.079515/s1.
